# Easy-to-Apply Hydrogel Patch for Field Treatment and Monitoring of Equine Wounds

**DOI:** 10.3390/gels11050328

**Published:** 2025-04-27

**Authors:** María Emilia Zambroni, Patricia A. Bertone, Ana Lucía Cabral, Andrés S. Boatti, Silvia V. Romanini, Sol R. Martínez, María Lorena Gómez

**Affiliations:** 1 Instituto de Investigaciones en Tecnologías Energéticas y Materiales Avanzados (IITEMA), Universidad Nacional de Río Cuarto and Consejo Nacional de Investigaciones Científicas y Tecnológicas (CONICET), Campus Universitario, Río Cuarto 5800, Argentina; mzambroni@exa.unrc.edu.ar; 2 Departamento de Clínica Animal, Facultad de Agronomía y Veterinaria, Universidad Nacional de Río Cuarto, Campus Universitario, Río Cuarto 5800, Argentina; pbertone@ayv.unrc.edu.ar (P.A.B.); aboatti@ayv.unrc.edu.ar (A.S.B.); 3 Departamento de Patología Animal, Facultad de Agronomía y Veterinaria, Universidad Nacional de Río Cuarto, Campus Universitario, Río Cuarto 5800, Argentina; sromanini@ayv.unrc.edu.ar

**Keywords:** wound healing patches, hydrogel, collagen, torn wound, equine

## Abstract

The cicatrization process, which is critical to equine health, directly affects overall well-being by preventing infection, minimizing tissue damage, and restoring optimal function. Herein, we present a case of a 5-year-old sorrel mare with a torn skin wound on the dorsal aspect of the metatarsal region of the left hind limb, treated locally with an antibiotic-free transparent hydrogel-based patch while monitoring its healing process. The patch induced pink granulation tissue in the treated area after 42 days, while keloid formation was observed in the untreated area. Wound measurements showed a reduction over time with patch treatment, with complete healing achieved at 116 days. Capillary formation and a velvety appearance were observed on day 80. Histological analysis revealed mature granulation tissue, fibrocyte formation, abundant capillaries, organized collagen fibrils, and development of type III collagen in the treated area. Interestingly, no inflammatory response was observed during treatment. The hydrogel patch not only accelerated healing, but also controlled excessive granulation tissue formation. This treatment represents an innovative approach to equine wound management that updates applications for owners while reducing costs.

## 1. Introduction

The main challenge in skin regeneration research is developing bioactive materials that promote wound healing [1,2]. This complexity stems from the skin structure and its multiple phases of the healing processes, which involve a plethora of biological pathways, cellular responses, and environmental factors [3]. Successful dermatologic tissue reconstruction requires not only materials that accelerate cellular proliferation, but also those that mitigate infection risks and reduce inflammation. Current knowledge emphasizes that achieving optimal results in dermatologic tissue reconstruction requires a well-suited dressing for the affected area [4,5,6]. In this regard, Liang et al. outlined four key requirements for effective dermatologic wound dressings: (1) excellent tissue compatibility, ensuring that the material does not induce adverse immune responses or cytotoxic effects; (2) adequate moisture retention, which maintains a hydrated environment conducive to cell migration and tissue regeneration; (3) appropriate physical and mechanical properties, allowing the dressing to adapt to the wound’s shape, maintain structural integrity, and provide protection; and (4) compatibility with surface and biochemical properties; promoting optimal cell adhesion and migration [7]. These properties play an important role in reducing side effects at the injury site, thereby preventing external infection. Moreover, they facilitate exudate absorption and stimulate cell proliferation in the wound area, ultimately promoting more effective scarring [8].

Wound healing is a biological process influenced by various factors that can significantly affect its progression. Factors such as wounds located in highly mobile areas, extensive tissue damage, and pathogenic bacterial growth can all contribute to delayed or impaired healing outcomes [9,10]. These complications are particularly common in challenging cases where conventional healing processes are insufficient. As a result, secondary intention healing is often required, a process in which wounds are left to heal gradually from the bottom up through the formation of granulation tissue, contraction, and epithelialization [11,12]. To boost healing outcomes, moist wound healing has emerged as an effective treatment [13]. This method maintains an optimal environment that promotes cell migration, enhances autolytic debridement, and minimizes the risk of infection. The development of hydrogel dressings has revolutionized this approach by providing a versatile solution capable of retaining moisture while also promoting oxygen exchange and delivering bioactive agents directly to the wound site [14,15]. Recent advances in wound care have highlighted the potential of hydrogel-based dressings as effective instruments to promote tissue regeneration, particularly in complex or chronic wounds [16,17]. Hydrogels composed of methacrylic acid (MAA), hyaluronic acid (HA), and collagen have garnered increasing interest due to their inherent biocompatibility, their capacity to sustain a moist healing environment, and their ability to emulate extracellular matrix components [18,19].

Furthermore, light-curing formulations offer several advantages, including precise control over gelation, customizability in shape and size, and preservation of bioactive agents [20]. These features are of particular relevance in the field of veterinary medicine, where the treatment of extensive and irregular wounds, such as those of equine patients, demands adaptable and robust materials [21,22]. Recently, reports underscored the growing importance of these multifunctional hydrogels in clinical practice, emphasizing the need to assess their performance in real-life scenarios [10,23].

The process of scarring, or wound healing, is particularly important in horses due to their unique physiological characteristics, their reliance on mobility for overall health, and the distinct challenges associated with their anatomy, behavior, and living environment [24]. Impaired healing in horses can lead to chronic wounds, reduced performance, and increased susceptibility to secondary infections, ultimately compromising their quality of life. The size and strength of horses complicate wound management and require careful handling to ensure both the safety of the veterinary team and the well-being of the horse [25]. Additionally, the natural flight response and heightened fearfulness of horses can further hinder effective wound care, underscoring the importance of understanding equine behavior. Environmental factors also present challenges, as outdoor conditions often expose wounds to potential contaminants, increasing the risk of infection. Moreover, equine wounds frequently occur in areas prone to constant movement or weight-bearing, which poses further obstacles to effective dressing and immobilization [26]. Consequently, implementing tailored wound management strategies that prioritize infection control, address anatomical complexities, and consider behavioral factors is crucial to ensuring successful recovery and maintaining the horse’s functional capacity [27].

In the equine lifestyle, which relies heavily on physical prowess, rapid and efficient wound healing is critical to maintaining performance and preventing complications. Horses are often involved in athletic activities such as racing, show jumping, and endurance riding, where injuries are common and can significantly impact their ability to perform [28]. Consequently, effective wound management is essential not only for the horse’s well-being but also for ensuring their long-term usability in competitive or working environments. A comprehensive study conducted by Theoret et al. highlighted a concerning trend: a significant proportion of equine wounds are managed directly by owners without professional veterinary guidance. This lack of informed intervention has contributed to wounds being identified as the second leading cause of equine mortality, highlighting the need for improved educational resources and accessible treatment methods for equine caretakers [29].

The purpose of this case study is to contribute to the development of a straightforward and effective method for promoting equine wound healing. By investigating practical strategies that owners can implement independently, this study aims to provide insights that enhance understanding and improve treatment approaches. Empowering owners with an effective tool for wound management holds the potential to reduce the dependence on clinical intervention, minimize treatment costs, and ultimately improve outcomes for horses. By equipping caregivers with accessible solutions, this initiative strives to bridge the gap between professional care and practical home-based management strategies, thereby facilitating a more proactive approach to equine wound care.

An important feature of the patch employed in this work is its transparency, which enables real-time visual monitoring of the wound, allowing caregivers and veterinarians to assess the healing progress without disturbing the affected area. This feature is particularly beneficial where frequent dressing changes can disrupt the healing process and increase the risk of infection. In contrast, conventional dressings such as opaque hydrocolloid and foam-based dressings require regular removal for wound assessment, which can result in mechanical stress to the regenerating tissue and potential contamination. Recent studies have highlighted the need for transparent wound dressings to improve wound observation while maintaining a moist healing environment [30]. However, many of these materials suffer from poor mechanical stability, limited antimicrobial properties, or require the employment of cost nanoparticles. Our silsesquioxane-based hydrogel patch overcomes these limitations by providing both structural integrity and intrinsic antimicrobial activity, making it a superior alternative for advanced wound management applications [31,32].

The flexibility of our material ensures that the dressing adapts to different anatomical areas, including challenging regions prone to movement or pressure. The adhesive properties of the patch provide secure attachment to the skin, even in active horses, minimizing the risk of detachment or contamination. In addition to being transparent and flexible, the dressing exhibits hemostatic properties that help control bleeding and promote faster clot formation, which is highly relevant in minimizing blood loss from acute injuries. Furthermore, the patch is designed to support the natural healing process by maintaining a moist environment conducive to cellular regeneration and tissue repair. Its confirmed antiseptic benefits help reduce bacterial load at the wound site, lowering the risk of infection and accelerating recovery [31,32]. This combination of features makes the patch a versatile and effective solution for equine wound management, addressing key challenges such as mobility, environmental exposure, and infection control.

Finally, although this study analyses a single clinical case, the experimental design ensures the reliability of the results by using the same animal as both the control and treated subject. This approach minimizes intra-individual variability because each site on the animal serves as its control, thereby reducing the influence of biological susceptibility and increasing the robustness of the observations. Previous studies have shown that within-subject comparison models are effective in wound healing research because they provide more consistent assessments of treatment efficacy while limiting the need for large sample sizes [33]. In addition, this method is consistent with the 3Rs (Replacement, Reduction, and Refinement) principles of animal research, ensuring ethical and scientifically sound experimentation [34]. These considerations strengthen the validity of our findings and support the potential clinical relevance of our material.

In summary, developing effective bioactive wound dressings for skin regeneration, particularly in equine models, is challenging. Optimal materials must balance cellular proliferation, infection control, and inflammation reduction, while addressing species-specific biomechanical and environmental factors. This study presents a versatile dressing designed to enhance healing outcomes by integrating tissue compatibility, moisture retention, and mechanical resilience for equine treatment.

## 2. Results and Discussion

### 2.1. Synthesis of the Hydrogel Patch

Manufacturing the patch for the equine treatment requires a tailored approach capable of producing patches in different shapes, allowing precise adaptation to a specific wound site. In this study, a visible light photopolymerization technique was employed to synthesize the hydrogel patch over an adherent film to ensure its suitability for its clinical applications. The successful polymerization of the hydrogels under air-equilibrated conditions highlights the efficiency of the chosen photoinitiator system [31,35].

**Figure 1:**
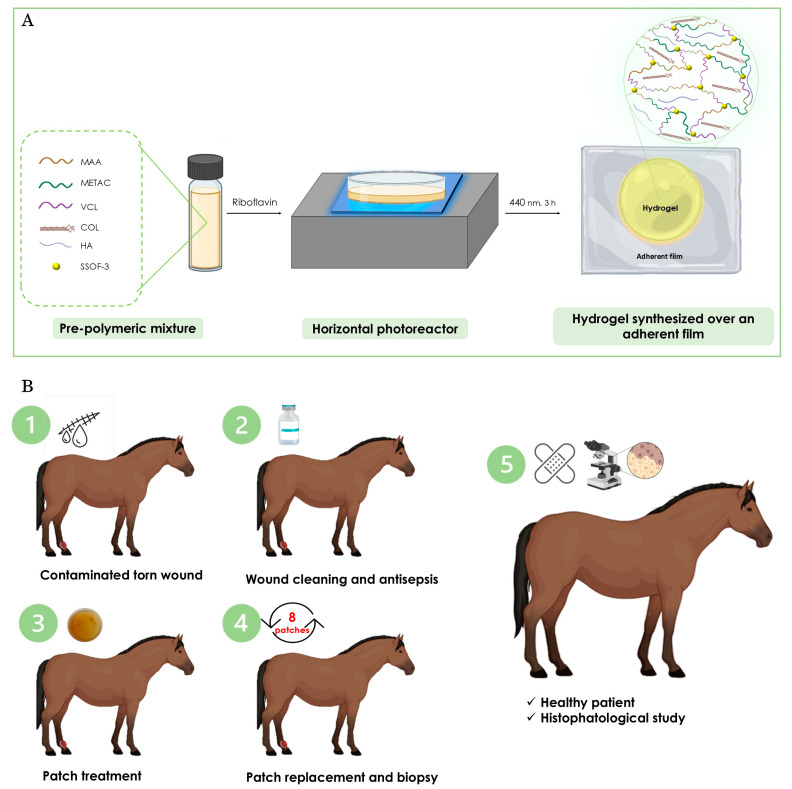
Schematic representation of (**A**) patch fabrication and (**B**) summary of the treatment of the mare’s torn wound.

**Figure 2:**
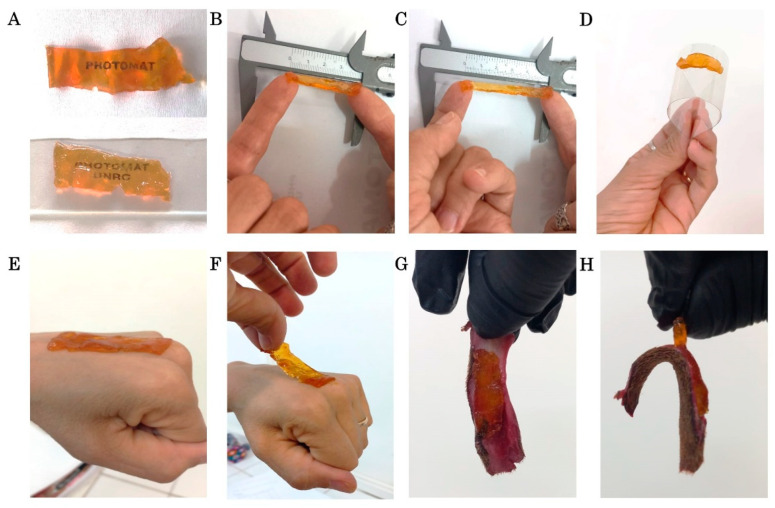
Photograph illustrates the primary characteristics of the material. From left to right, in the top row, (**A**) shows transparency and (**B**–**D**) flexibility. The bottom row, from (**E**) to (**F**), illustrates the adhesion properties of the patch used to treat the mare’s wound on human skin and on (**G**,**H**) equine skin.

The material used is a photopolymerized hydrogel patch containing MAA, VCL, and METAC in its composition (Figure 1A); details of the synthesis are in the experimental section. In addition, COL and HA were integrated into the formulation and tightly assembled within the gel matrix during the manufacturing process [31,32]. As delineated in Figure 1B, a schematic representation of the treatment of the mare’s torn wound is provided. This subject will be discussed in detail in the subsequent section. The transparency, flexibility, and adherence of the resulting COL-HA dressing are illustrated in Figure 2A–D. The combination of dressing transparency and adherent film facilitates veterinary handling and enables unobstructed monitoring of the wound site. This feature has the potential to reduce the time required for bandage changes, decrease treatment costs, and minimize unnecessary inconvenience to the animal.

Transparency is a critical aspect for monitoring the progress of an injury, yet it remains an unavailable feature in the majority of commercially available patches. Conversely, flexibility and strong adhesion are essential for effective bandage application, particularly in mobile areas, such as the case report presented here [5]. Figure 2D–H clearly shows the adhesion of the patch to acetate, human skin, and equine skin.

### 2.2. Application of the Hydrogel Patch in the Treatment of a Full-Thickness Wound in a Mare

The clinical potential of the developed hydrogel patch was explored through a clinical report that involved the treatment of a large, full-thickness cutaneous wound in a mare. This real-world application enabled the evaluation of the dressing’s biocompatibility, ease of use, and therapeutic efficacy under veterinary conditions. The subsequent section outlines the treatment protocol and the progression of the wound through the healing period. A schematic representation of the treatment is presented in Figure 1B.

**Figure 3:**
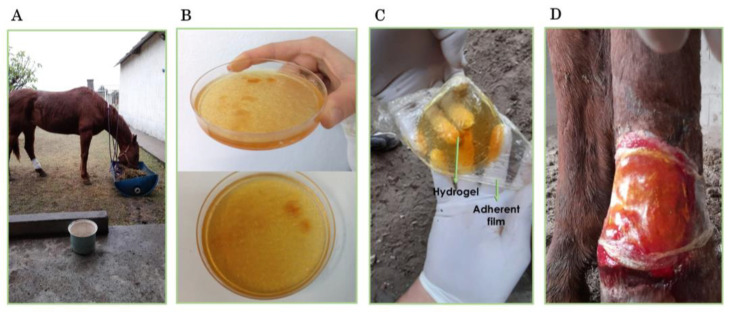
The images in this set include those of the animal, the materials used, and the treated wound. Panel (**A**) documents the adult mare with a tear in the hind limb treated with the patch, panels (**B**,**C**) show the applied patches, and (**D**) depicts the animal at the beginning of the treatment with the patch.

As illustrated in Figure 3A–C, images of the mare patient and the synthesized hydrogel patches are shown. Figure 3D displays the treated wound area of the animal with the patch at the beginning of the treatment period. As previously mentioned, the transparency of the material allows the monitoring of the wound aspect to prevent undesirable changes in appearance or color, or the detection of an incipient infection. This is a noteworthy characteristic of this particular type of patch, which is an uncommon feature among equine bandages.

**Figure 4:**
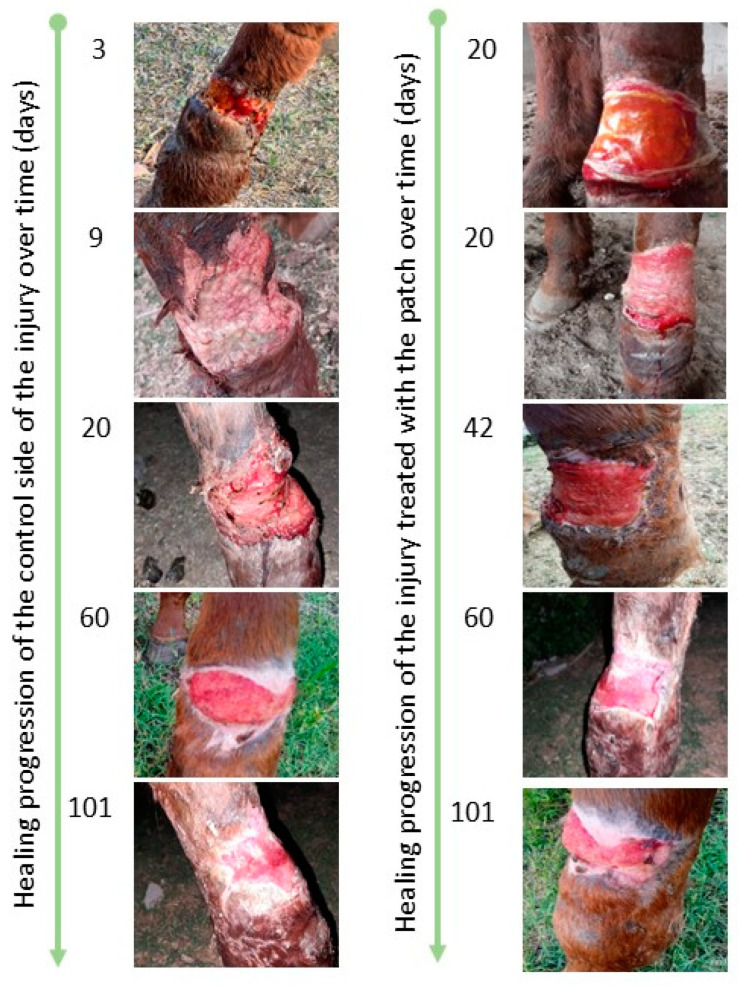
Photographic progression of the wound healing in an adult mare. (**Left**): medial zone (Control), (**Right**): lateral zone where the patch was applied (Treated).

After the tear, clinical management of the wound began. Figure 4 shows the appearance of the contaminated open wound bed and irregular edges, 20 cm × 10 cm high, covering 75% of the anatomical area on day 3 after the injury. On day 9 post-injury (left panel in Figure 4), the wound showed clear signs of infection under initial treatment with nitrofurazone 0.2%. On the tenth day following the injury, the hydrogel patch was administered as the sole treatment on the lateral side of the lesion, while the medial side of the lesion was left untreated. The initial dressing change with the hydrogel patch occurred after 20 days. The lateral side exhibited notable improvement with bleeding under the hydrogel patch during the change, signifying substantial progress on the treated side. The right segment of Figure 4 illustrates the appearance of the wound at day 20, with granulation tissue on the treated side.

As shown in Figure 4, 60 days after the beginning of treatment, the lateral side treated with the hydrogel patch had a velvety appearance indicative of granulation tissue. In contrast, the untreated medial side displayed the presence of fibrous tissue, characterized by a pearly appearance. On day 101, observations showed hypertrophic healing on the untreated medial side of the left hind limb, in contrast to the lateral side treated with the hydrogel patch, which showed uncomplicated healing. Final scarring was observed on day 116. These results are particularly encouraging when compared to the prolonged healing time of over 40 days in a small 6.25 cm^2^ full-thickness equine wound model treated with a special peptide-conjugated collagen–chitosan hydrogel [11]. Moreover, the literature on equine wound healing indicates that smaller, uncomplicated wounds (approximately 2 to 30 cm^2^) on the distal limbs typically heal within 34 to 51 days, depending on the type of treatment [36]. However, larger wounds, particularly those that are infected or located on the distal limbs, tend to require longer healing times due to factors such as reduced vascularity, increased motion, and a higher likelihood of complications, including exuberant granulation tissue.

Although specific studies reporting healing times for infected wounds exceeding 100 cm^2^ are limited, it is reasonable to expect significantly prolonged recovery compared to smaller wounds. The 116-day healing period observed in our case is consistent with expectations for large, infected wounds treated with conventional dressings, suggesting that the hydrogel patch provided a healing environment comparable to standard treatment approaches.

The findings attained using this material for secondary healing of equine wounds demonstrate notable efficacy and competitiveness when benchmarked against alternative treatment options. This is particularly noteworthy considering that the entire treatment was performed in the horse’s environment, without any additional clinical assistance [36,37,38].

**Figure 5:**
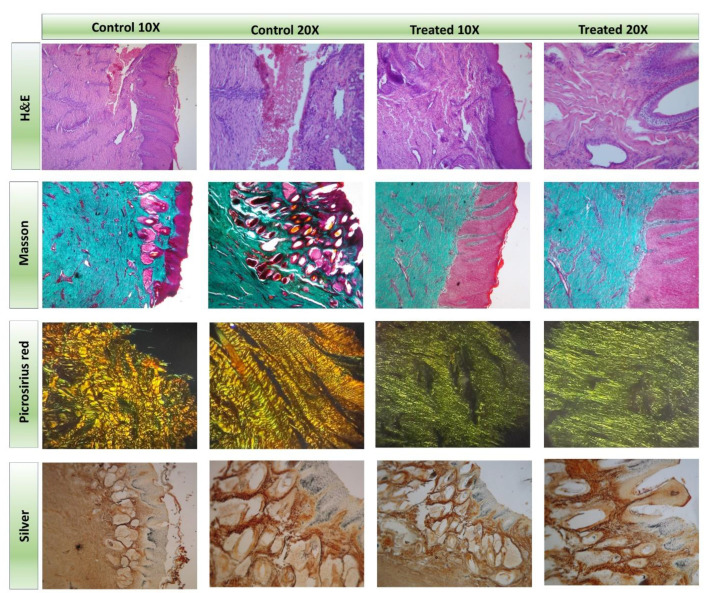
Histological sections from top to bottom: H&E, Masson’s Trichrome, and Picrosirius Red and Silver stains performed on the control (**Left**) and treated (**Right**) sides of the injury at 10× and 20×.

Skin biopsies were taken from both the control and treated tissue on day 80 to further evaluate the effect of the patch on the scarring process. Histological examination, including staining with H&E, Masson’s trichrome, Picrosirius red, and Silver stains, was performed and is summarized in Figure 5.

The H&E staining of the treated tissue reveals a well-ordered arrangement of collagen fibers (first row–last column in Figure 5) that respects the natural tissue architecture, accompanied by the presence of fibroblasts and fibrocytes. Conversely, the control tissue displayed collagen fibers in the dermis with no discernible organization (first row–first and second columns in Figure 5). Masson’s trichrome staining highlights a softer tone in the collagen fibers of the treated tissue compared to the control, indicating a more refined composition. Furthermore, the treated area showed a higher ratio of fibroblasts to fibrocytes, along with a more organized and abundant fiber arrangement (the last two columns in the second row of Figure 5).

Picrosirius Red staining reveals a predominant green hue in the treated tissue, demonstrating the prevalence of type III collagen (last two columns of the third row in Figure 5), which is characterized by fine fibers and a significant presence of reticulin. The control tissue shows a predominance of yellowish color corresponding to type I collagen fibers (first and second columns of third row in Figure 5). Notably, while type I collagen is the primary component of normal skin, its prevalence in the control tissue aligns with the characteristics of keloid-like scarring observed in macroscopic images. The results indicate increased collagen production in the patch-treated tissue, underscoring the beneficial effects of the treatment on tissue regeneration. The enhanced presence of type III collagen likely contributes to the accelerated healing process observed, highlighting the potential of the hydrogel patch as an effective treatment in equine wound healing.

Finally, Silver staining of the treated tissue shows collagen fibers in the dermis with milder staining, highlighting the intricate arrangement and architecture of the tissue (last two columns of last row in Figure 5). In contrast, the control tissue shows heavily stained collagen fibers, indicating a lower level of organization.

### 2.3. Final Remarks

The hydrogel developed in this study incorporates key biocompatible materials such as MAA, VCL, METAC, COL, and HA, which contribute to its dual role of infection prevention and wound healing. MAA enhances antimicrobial efficacy thanks to its acidic nature, which destabilizes bacterial cell membranes and creates an unfavorable environment for microbial growth. VCL exhibits thermoresponsive behavior, allowing for improved conformability and controlled drug release at physiological temperatures. METAC, a cationic monomer, facilitates bacterial precipitation because the charge disrupts the bacterial membrane potential, resulting in cell death and inhibiting biofilm formation.

In addition to its antimicrobial properties, the hydrogel matrix contributes significantly to wound healing by providing a bioactive environment that supports tissue regeneration. COL acts as a structural scaffold that closely mimics the native extracellular matrix, facilitating key cellular processes such as adhesion, migration, and proliferation, which are critical for the formation of new granulation tissue and epithelialization. HA complements this activity by maintaining a moist wound environment, reducing local inflammation and promoting angiogenesis, thereby accelerating tissue repair. The synergy between these components promotes an optimal microenvironment that favors both microbial control and regenerative healing.

Importantly, histological analysis of the regenerated tissue revealed the presence of type III collagen, a marker typically associated with cartilage structures, but also indicative of extracellular matrix remodeling in the early stages of deep dermal wounds. This finding suggests that the hydrogel not only promoted structural regeneration, but may have influenced matrix organization and quality, contributing to more robust and functional tissue repair. Taken together, these results highlight the dual functionality of the hydrogel and confirm its therapeutic potential to promote effective and biologically active wound healing, as demonstrated in this equine clinical case.

This study underscores the potential of the hydrogel patch as a promising candidate for the treatment of complex skin wounds. The combination of tailored physicochemical properties, antimicrobial activity, and conformability to irregular wound sites demonstrates the translational relevance of the material. The potential applications of this hydrogel could be extended beyond equine wound healing and have promising implications for human wound care, burn treatment, and chronic wound management. Future studies will focus on in vivo evaluations with larger sample sizes to validate its efficacy, optimize formulation parameters, and explore its combination with additional bioactive agents such as antimicrobial peptides or growth factors to enhance its therapeutic potential. In addition, scaling up the manufacturing process and evaluating long-term stability are essential steps to move this multifunctional hydrogel into clinical and commercial areas.

Despite the promising outcomes observed, further supported by the comparative response in the untreated control area of the same animal, one of the primary limitations of this study is the lack of a statistically significant sample size. As the evaluation is based on a single clinical case, the findings, while illustrative, limit the ability to generalize the results to broader populations or clinical conditions.

## 3. Conclusions

This study evaluated the therapeutic efficacy of a hydrogel patch composed of MAA, VCL, METAC, COL, and HA in the treatment of a large equine laceration without the use of conventional antibiotics. The transparency of the patch allowed for non-invasive monitoring and control of the wound, contributing to a significant improvement in both the rate and quality of healing of an extensive injury. In particular, the patch effectively prevented keloid formation by promoting the intrinsic production of type III collagen, as demonstrated by histologic analysis.

In summary, the hydrogel patch demonstrated the dual benefit of minimizing microbial contamination, a critical factor in wound healing, particularly in rural areas, and facilitating connective tissue proliferation. The increased production of type III collagen played a key role in reducing the risk of fibrotic healing complications. These findings establish the hydrogel patch as a practical and effective therapeutic option for treating lacerations in mobile anatomical regions of horses in their natural environment. In addition, its use provides a cost-effective alternative for equine wound care, reducing the need for intensive and costly clinical interventions and facilitating monitoring and control.

## 4. Materials and Methods

### 4.1. Materials

Methacrylic acid (MAA-98%), N-Vinyl caprolactam (VCL-98%), and [2-(methacryloyloxy) ethyl] trimetammonium (METAC-75%) were supplied by Sigma-Aldrich (St. Louis, ML, USA) in the as-received condition. Type I hydrolyzed collagen (COL) and Hyaluronic acid (HA—average Mw 25,000) were purchased from Natural Whey Supplementos, Argentina. Double-distilled water was further purified by an ELGA PURELAB Classic UV system (17 MΩ/cm) to remove ions and organic and particulate matter (0.2 μm filter).

### 4.2. Synthesis of Hydrogel Patch

The development of dermatological dressings containing MAA, VCL, and METAC as monomers and COL-HA to accelerate wound healing was reported in a recent study [31]. Our previous in vivo experiments employing rabbits as an animal model focused on three specific dressings, excluding those with COL and HA, as a full dressing containing hops was tested for its antimicrobial properties. However, the inclusion of hops delayed the healing process. Conversely, dressings with COL and HA demonstrated superior performance in in vitro wound healing assays [31]. In this work, we tested this material in a clinical case involving a large, challenging torn wound on an equine.

#### 4.2.1. Pre-Polymeric Mixture Preparation

The hydrogel precursors were prepared by dissolving MAA, METAC, and VCL, used as monomers, with a total concentration of 50% *w*/*w* relative to the solvent (H_2_O). The proportion employed for each monomer, described in molar concentration, was: 0.79 M for MAA, 1.22 M for VCL, and 1.62 M for METAC. COL, HA, and the crosslinker were added in concentrations of 10% *w*/*w*, 1% *w*/*w*, and 2% *w*/*w* relative to the total monomer mass, respectively, in the biocompatible aqueous medium. The prepolymeric solution contained a hydrophilic polymer backbone to ensure moisture retention and mechanical stability, a photoinitiator composed of Riboflavin and a silsesquioxane (SSO-3) to initiate polymerization upon exposure to light, and enhanced antimicrobial and regenerative properties, as demonstrated in a previous work [32]. The formulation was homogenized under continuous stirring at room temperature until complete dissolution of all components was achieved.

#### 4.2.2. Photopolymerization Process

The prepared pre-polymeric solutions were poured into specially designed molds to achieve the desired shape and thickness of the dressings. An adhesive film was incorporated that extended beyond the 90 mm diameter Petri dish. Figure 1A illustrates the reactants and fabrication procedure. The pre-polymeric sample was poured onto this film, and the dish was sealed and irradiated for 3 h in a horizontal photoreactor to ensure complete monomer conversion [14]. In the pre-polymer mixture, riboflavin was the only compound capable of absorbing blue photons from the irradiation source, confirming its role as the primary photoinitiator.

Polymerization was initiated by exposing the formulations to a blue light source (wavelength: 450–470 nm and ~30 mW/cm^2^) in a controlled environment. The light source was positioned at a fixed distance to ensure uniform exposure and consistent cross-linking throughout the hydrogel matrix. The irradiation chamber was maintained at room temperature and air-equilibrated conditions to prevent oxygen inhibition and ensure complete polymerization. No degradation or degradation of the incorporated reactants was observed during the photoreaction process.

#### 4.2.3. Post-Processing and Characterization

Following polymerization, the material was stored in the dark for 24 h, and the patch supported over the adherent film was carefully removed from the molds and thoroughly washed with sterile distilled water to remove any unreacted monomers. The resulting patches were flexible, transparent, and retained a high moisture content, which is critical for wound healing applications. The mechanical properties, swelling capacity, and biodegradability of the hydrogels ensure optimal performance for clinical use, as previously demonstrated [31]. The dressings were then sterilized under UV light for 20 min, sealed with parafilm^®^, and stored in a dark, dry place until use. A total of 8 patches were synthesized according to the above protocol.

### 4.3. Case Report

A 5-year-old sorrel mare, weighing 430 kg and used for recreation and riding, was reported to the veterinary medical service from a farm east of the town of Elena, Córdoba province, Argentina (32°34′54″ S 64°23′03″ W). The equine had a torn skin wound on the dorsal aspect of the metatarsal of the left hind limb. The injury had occurred the day before the consultation and was caused by the traction of a wire when the animal attempted to jump between paddocks. Informed consent for treatment was obtained from the owner.

The in vivo wound healing experiments were conducted in accordance with a protocol approved by the Research Ethics Committee of Universidad Nacional de Rio Cuarto (Reg. Num. 429/22) and in accordance with international standards. The clinical study adhered to the 3Rs principle (Replacement, Reduction, and Refinement) and was conducted with the signed consent of the horse’s owner. A schematic representation of the whole treatment is presented in Figure 1B.

#### 4.3.1. Clinical Findings

Upon clinical examination, the animal presented with grade 2 out of 5 claudication of the left hind limb. The torn wound was infected and exhibited extensive skin loss on the dorsal aspect of the metatarsal, corresponding to the hind limb.

Examination of the wound revealed skin injury without muscle or tendon involvement. No other general abnormalities were noted on physical examination; temperature, mucous membranes, and capillary filling were within normal limits. No changes were observed in the hemogram.

#### 4.3.2. Diagnosis

The wound had irregular edges, measuring 8 cm × 16 cm, and covered 75% of the left metatarsal region. It involved a tear of the skin and subcutaneous cellular tissue, without affecting the extensor digitorum longus and extensor digitorum lateralis tendons, but with visualization of bone tissue.

#### 4.3.3. Treatment

The treatment was performed in the animal’s natural environment. The treatment involved antibiotic treatment with Ceftiofur Sodium (2 mg/kg) IM over 5 days, accompanied by antisepsis of the wound with Chlorhexidine 4% (Soapy Sol. Cream, IQB^®^, Córdoba, Argentina) with neuroleptoanalgesia of the animal: Detomidine (0.02 mg/kg, EV, Echimidine 1%. Over^®^ and Butorphanol (0.03 mg/kg EV, Torbutrol 1% Zoetis^®^, Parsippany-Troy Hills, NJ, USA) and placement of gauze with Nitrofurazone 2% (Cream, IQB^®^, Argentina) and bandages every 2 days, without obtaining results.

On the tenth day, the treatment was changed to the exclusive use of the hydrogel patch [22], which was replaced every 10–12 days. The wound was stratified into two zones: the lateral, where the patch was applied (Treated zone), and the medial, without the patch (Control zone). The entire limb was covered with film. Wound size was measured and photographed. On day 80, a skin biopsy was taken from both the treated and control areas for histopathologic examination.

### 4.4. Histopathology and Histochemistry

The samples were evaluated using Hematoxylin/Eosin (H&E), Masson’s Trichrome, and Picrosirius Red and Silver stains [39,40,41]. Tissue samples were obtained with a scalpel from the treated and control zones. The samples were fixed with formaldehyde diluted in 1/10 buffer solution. Subsequently, the samples were cut into 5 μm-thick sections using a microtome. The prepared sections underwent dehydration cycles, paraffin embedding, and followed the standard histological procedures for H&E staining. Differential stains such as Masson’s Trichrome and Picrosirius Red and Silver stains were also performed for cellular characterization during skin healing. Photographic records were taken with a Zeiss Axio star microscope and a Canon G5 camera at 20× magnification at UNRC, Rio Cuarto, Argentina. Picrosirius Red staining was observed by polarized light microscopy.

### 4.5. Statistical Analysis

As this study is a single clinical case report, formal statistical analyses were not performed; findings are presented descriptively with visual documentation, clinical observations, and semi-quantitative comparisons. Histological experiments were conducted in triplicate, with at least four images recorded per area for each sample.

## Data Availability

The data presented in this study are available on request from the corresponding author.
